# Treatment of striae albae with combination of fractional radiofrequency and topical tretinoin: A randomized controlled trial

**DOI:** 10.1007/s10103-026-04975-5

**Published:** 2026-07-31

**Authors:** Gabriela Lladó Grove, Katrine E Karmisholt, Peter A Philipsen, Peter Bjerring, Merete Haedersdal

**Affiliations:** 1 Department of Dermatology, Copenhagen University Hospital – Bispebjerg, Copenhagen, Denmark; 2 Department of Clinical Medicine, University of Copenhagen, Copenhagen, Denmark; 3 Department of Dermatology, Aalborg University Hospital, Aalborg, Denmark

**Keywords:** RCT, Randomized controlled trial, Striae albae, Fractional radiofrequency, Topical, Tretinoin, Combination therapy, Drug delivery, POSAS

## Abstract

Mature striae distensae, called striae albae, are common but treatment-resistant dermal scars. Fractional radiofrequency (FRF) can potentially act as transdermal permeation enhancer for topical tretinoin (TT). This study investigated FRF and TT as a combination treatment for striae albae. Twenty patients were included, each with four anatomically comparable areas that were randomized to (i) TT (ii) FRF (iii) combination FRF + TT and (iv) untreated control. Three treatment sessions were performed one month apart, and TT was also applied in-between. The change in striae from baseline to 20-week follow-up (Δ) was assessed using the Patient Observer Scar Assessment Scale (POSAS, SUM: 6–60). At baseline, patients assessed the striae as moderate-severe (POSAS-PT: SUM medians 24–25) and at follow-up, they reported an improvement (POSAS-PT: ΔSUM 5–10 points), but only the combination treatment improved significantly better (FRF + TT vs. control *p* = 0.029). A blinded dermatologist confirmed a tendency towards treatment improvement (POSAS-OBS: ΔSUM: 0–2 point, all treatments vs control p≥0.269). A subtle tendency was observed in objective measures with 3D photographs. Combination treatment with FRF and TT was tolerable and improved patient-reported striae compared to control. Striae albae remain challenging, but the findings highlight a potential for future combination treatment approaches.

**Trial registration number:** NCT05461755

**Date of registration:** 15.07.2022

## Introduction

Striae distensae are very common, undesirable dermal lesions, that generally cause dissatisfaction among those affected. They typically occur after stretching of the skin, and mainly on the abdomen, buttocks, thighs and upper arms. Stretching of the skin is common during puberty growth spurts, pregnancies, in obesity, as well as extensive bodybuilding, but striae can also develop in patients with skin diseases or other conditions treated with oral or topical corticosteroids [1]. The emergence of striae initially presents itself in overstretched areas with linear, purple-reddish skin lesions called striae rubra. Over time, they fade to a paler, whitish color and exhibit scar-like epidermal atrophy known as striae albae [2]. In their striae rubrae phase, striae are easily identified by the red color component which also provides a straightforward target for treatment. In contrast, in mature striae, striae albae, the treatment targets are the remaining structural and atrophic components, which are extremely challenging to treat. Multiple treatment modalities for striae have been investigated, however, with limited and alternating effects [3], and as such there is an unmet need for alternative treatment approaches.

A variety of topical treatments have been investigated for both prophylactic and therapeutic use. Topical retinoids are recognized for their ability to stimulate reepithelization and fibroblast production, and topical tretinoin (TT) primarily, has been tested for striae in several studies [4, 5]. Generally, studies have found a possible effect of TT on striae rubrae after daily use for > 3 months, but with less convincing results for striae albae [6, 7]. In general, higher concentrations (0.1%) of tretinoin have been associated with improved outcomes, however, the higher concentrations may in turn increase the risk of adverse effects, especially with daily use for a longer period. The adverse reactions in the skin, such as itching and erythema, can significantly decrease compliance for indicated long-term use. Low-dose tretinoin (0.025%), on the other hand, has not been proven to have an effect on striae [8]. Other investigated topical substances include silicone gel and a wide variety of natural oils generally utilized with minimal adverse effects [9–11], however, lacking evidence of significant effect.

Mechanical and energy-based treatments include microdermabrasion and microneedling, and lasers and radiofrequency respectively [5, 12], all of which are widely utilized to improve the appearance of a variety of skin lesions. Targeting the early erythematous component in striae rubrae with vascular lasers has proven successful, while significant improvement with laser treatment of the structural components of striae albae has proven harder to achieve. Furthermore, laser treatments can be painful, cause considerable downtime, and present with side effects such as significant pigmentation changes [13]. Fractional radiofrequency (FRF) has generally proven to be a relevant tool for remodeling and tightening of the skin surface, since it is able to target the dermis [14], and is generally linked to reduced procedural pain, downtime, and risk of pigmentation change in comparison to lasers. Altogether, however, there is currently a lack of evidence for optimal treatment of striae albae, where improvement relies on targeting the structural components.

In the past decade’s development in the field of device-assisted topical delivery, an increasing interest in combination therapies for skin conditions has arisen [15, 16]. The use of energy-based devices and a topical drug in combination has the potential of a synergistic effect as the drug uptake can be enhanced significantly. While the technique is widely proven with laser, it has recently been shown that FRF also has potential to enhance transdermal permeation of a topically applied drug in a pre-clinical study [17]. A multitude of topical compounds have been investigated for device-assisted topical delivery, but the field leaves a wide range of potential combinations yet to be explored [16]. FRF and TT have individually been investigated and shown capabilities for improvement of the skin surface. The combination of FRF and TT has been investigated to some extent in prior studies that support the combinations’ potential, but with significant study limitations [7, 17, 18] The aim of this study was to explore the potential of FRF and high-dose TT (0.1%) combination therapy for striae albae, specifically, in a randomized, controlled and GCP-monitored clinical trial. This was conducted on the basis of a pre-clinical paper [17] on the specific FRF-device that verified its potential for increased topical uptake measured by fluorescence. A translational approach was applied combining a pre-clinical scientific basis with a literature background on device-assisted topical delivery principles, known properties of FRF as well as the body of evidence on high-dose topical tretinoin as potential combination treatment for striae albae. Highest tretinoin dose was chosen in order to maximize the chances of a positive outcome.

## Materials and methods

### Design

This was a prospective, randomized, within-subject controlled trial of mature striae distensae allocated to treatment with (i) TT alone, (ii) FRF alone, (iii) combination treatment with FRF and TT, compared with (iv) untreated control. Randomization was secured using a computer-generated block randomization list, and allocation concealment consisted of sealed opaque envelopes. Three monthly in-office treatments were provided (week 0, 4, 8), and 12 weeks after the 3rd treatment (week 20), the patients were evaluated at a follow-up visit.

The study was carried out at the Department of Dermatology, Copenhagen University Hospital, Denmark, between 4th quarter 2022 and 3rd quarter 2023. The trial was GCP-monitored, conducted in accordance with the Helsinki Declaration, and approvals from the Danish Medicines Agency (EudraCT: 2021–003153−39), the Regional Health Research Ethics Committee in Copenhagen (H-21038853) and the Danish Data Protection Agency (P-2021-686) were acquired. Informed participation consent was obtained from all study subjects, and registration on ClinicalTrials.gov (NCT05461755, ID: SDRFTT, date: 15.07.2022) was completed before study inclusion. Separate informed consent forms regarding the publication of images for academic purposes were also secured.

### Study population

Adults with striae albae were included consecutively when meeting the following criteria: Fitzpatrick skin type I-III with striae albae ≥ 1 year of age and ≥ 4 lesions with a length of ≥ 2 cm each. Additionally, included women of childbearing potential presented negative u-hCG prior to study treatments and used a safe contraceptive method throughout.

Main exclusion criteria comprised serious medical conditions, skin disorders, tattoos, tanned skin or recent surgery in the treatment areas at baseline, treatment with Isotretinoin within the past 6 months, regular use of non-steroidal anti-inflammatory drugs, known allergies to interventional drug, metal implant in the treatment area or any electronic device. Pregnant or lactating women were excluded as well.

### Interventions

All treatments were performed by a single, trained clinician (GLG). After baseline evaluation, treatment allocation was randomized into four anatomically comparable areas with striae (1) combination FRF + TT, (2) TT alone, (3) FRF alone, and (4) untreated control. To avoid any bystander effect, the defined areas were separated by at least 0.5 cm skin surface. Each area was outlined on a fixed template in order to secure accurate continuity throughout the study. In 3 treatment sessions, FRF- and TT-allocated areas were treated in-clinic. The combination areas were first treated with FRF and TT was applied directly thereafter.

Patients were instructed to continue to apply TT equally on both TT-allocated areas at home, daily or as tolerated. The randomization was done in blocks that ensured that the two areas allocated to TT application were on the same anatomical side to avoid incorrect home-application.

#### Fractional radiofrequency

Treatment with FRF was performed with the CE-marked Venus VivaMD™ system (Venus Concept Inc.) with 80-pin tips which can deliver a maximal energy output of 124 mJ/pin. Treatment consisted of a single pass with multiple repositioning of the 20 × 8 mm tip depending on the size of treatment area with no overlap. A voltage of 280 volts and a pulse duration of 10 (5 subjects) or 20 (19 subjects) milliseconds at random were used. If deemed necessary, local anesthesia was applied with topical lidocaine and prilocaine (Emla 25 + 25 mg/g, Aspen Nordic) before FRF-treatment.

#### Topical tretinoin

The utilized topical was tretinoin 0.1% (Retirides 0,1%, Ferrer International) with a molecular weight of 300.4 Dalton. In-office, TT was applied by the treating clinician, and for home-use patients were instructed to ensure optimal and correct application. To decrease the risk of adverse effects, patients would apply the cream every other day during the first week – if tolerability was acceptable then the frequency was increased up to one application daily.

### Subject characteristics

Demographics included age, sex and body mass index (BMI). The anatomic site of included striae and a history of previous striae treatment were also recorded. The usage of tretinoin cream throughout the study was noted. Outcome measures and demographics were investigated for possible correlations.

### Main outcome measures

#### Patient-observer scar assessment scale (POSAS)

The main outcome measures were conducted as standardized on-site assessment of striae appearance at baseline and at 20-weeks follow-up on the clinically available Patient-Observer Scar Assessment Scale (POSAS, version 2.0), by patients, and a blinded dermatologist with scar assessment experience (KK), respectively. Each assessment comprises of a score based on multiple items (SUM) and an overall score (OVERALL).

The POSAS-PT is assessed by the individual patient at baseline and follow-up:

SUM (6–60 points): 6 items; *pain*,* itching*,* color*,* stiffness*,* thickness* and *irregularity* each on a scale of 1–10 (1: no/as normal skin, 10: yes/very).

OVERALL (1–10 points).: 1 item; opinion of skin lesion compared to normal skin on a scale of 1–10 (1: as normal skin, 10: very different).

The POSAS-OBS is assessed by the blinded dermatologist at baseline and follow-up:

SUM (6–60 points): 6 items; *vascularity*,* pigmentation*,* thickness*,* relief*,* pliability* and *surface area* each on a scale of 1–10 (1: normal skin, 10: worst imaginable scar).

OVERALL (1–10 points).: 1 item; opinion of skin lesion compared to normal skin on a scale of 1–10 (1: normal skin, 10: worst imaginable scar).

### Supporting measures

#### Exploratory visualization

Clinical images were collected with a digital camera and a 3D camera (Cherry Imaging, Yokneam, Israel) as supporting visualization of the striae development from baseline to 20-week follow-up. With the 3D camera, an automated filter of depression in the skin surface was used with yellow color demarcating increasing depth with increasing color intensity.

#### Tolerability

Procedure-related discomfort was assessed on a Numerical Rating Scale (NRS) in relation to the treatment session with FRF and by anatomic site. Common side effects to the interventions were assessed on a 4-item Likert Scale 0: None, 1: Light, 2: Moderate, 3: Severe. Additionally, there was monitoring of any unexpected side effects and serious adverse events throughout the study.

#### Patient satisfaction

Satisfaction for each intervention and control at the end of the study (20-week follow-up) was collected on a 5-item Likert Scale; Very unsatisfied, Unsatisfied, Neutral, Satisfied, Very satisfied.

### Statistics

Sample size was chosen based on a reasonable number of voluntary subjects with the approval of the local GCP unit due to the exploratory nature. The within-subject design led to a sample size four-fold the number of subjects.

Patient characteristics were displayed with descriptive statistics with medians and interquartile range (IQR) or absolute ranges.

Statistical tests were applied to the POSAS data with non-parametric tests of paired data (Wilcoxon signed-rank test). Corresponding plots with medians and *score distribution ranges* were presented for a selection of the data for illustrative purposes. Spearman’s correlation was applied for outcome measures in relation to patient demographics and treatment characteristics. Pearson’s chi-square test was used to test the association between pain and anatomic site.

A p-value of < 0.05 was considered statistically significant: Test to compare baseline with any follow-up visit for each treatment is presented as ‘p’-values, while ‘Δp’-values were calculated by comparing the change between the different treatments and the control for any given follow-up point. The change (Δ) was presented as an absolute score reduction from baseline to follow-up, which, based on the POSAS scale, corresponded to a reported/assessed improvement. For tests that compared all subgroups (e.g. 6 comparisons for each SUM score items), p-value significance was regarded under the relevant Bonferroni correction. SPSS Statistics for Windows v.29 was used for all statistical calculations (IBM, NY, USA).

## Results

### Subject and treatment characteristics

**Fig. 1:**
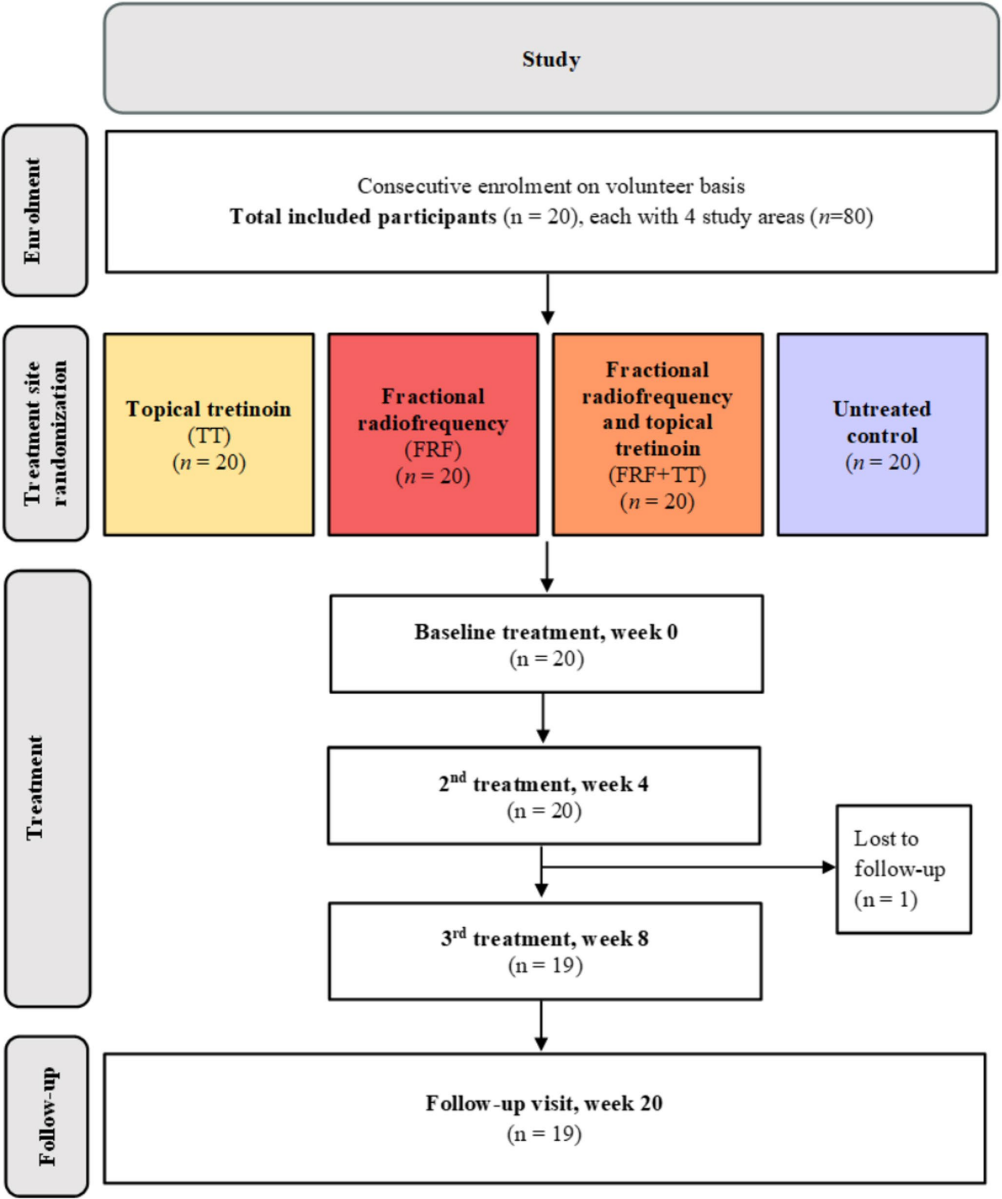
Flowchart of study, *n* = 20

Twenty subjects were enrolled comprising of a total of 80 included study areas. Nine-teen subjects completed the study. See Fig. 1 for study flowchart.

The vast majority of patients were women (95%), while only a single man participated (5%). The median age was 30.5 years (IQR: 27–37) with a range between 23 and 57 years, and the median body mass index was 24.0 kg/m^2^ (IQR: 21.2–26.4) with a range between 18.6 and 33.5. Patient skin type mainly comprised Fitzpatrick skin type II (55%), but skin types III (35%) and I (10%) were also represented.

The patients presented with striae in six different anatomical regions, nearly half of them from the abdominal region (45%), the rest in the buttocks (20%), inner thighs (15%), hips (10%), lower back (5%) and knees (5%). Only one of the included patients had previously undergone specialized treatment for striae on the abdomen with an energy-based device (pulsed dye laser), however, without any significant changes.

The usage of tretinoin cream throughout the study was a median of 17.2 g (IQR: 13.5–22.9) with a range between 4.3 and 27.2 g. Treatment area sizes were adjusted to the individually included areas and presented with a median area size of 46.5 cm^2^ (IQR: 32–62) with a range between 14 and 112 cm^2^. The largest areas were located on the abdomen while the smallest included treatment areas were on the inner thighs. See Table 1 for an overview.

**Table 1:** Characteristics of subjects and treatment

**Subject characteristics**	
Women, *n* (%)	19 (95%)
Age (years), median (IQR)	31 (28–37)
BMI (kg/m^2^), median (IQR)	24.0 (21.2–26.4)
Fitzpatrick Skin Type, *n* (%)	
I II III	2 (10%) 11 (55%) 7 (35%)
**Treatment characteristics**	
Tretinoin study usage (g), median (IQR)	17.2 (13.5–22.9)
Treatment area size (cm^2^), median (IQR)	46.5 (32–62)

### Main outcomes measures

#### Baseline POSAS scores

At baseline, patients assessed their striae in all 4 areas as moderate-severe on POSAS-PT SUM and OVERALL. Meanwhile, the blinded observer assessed the striae as mild on POSAS-OBS SUM and OVERALL. See Table 2 for an overview of POSAS-PT and POSAS-OBS SUM and OVERALL data.

The four randomization areas in each patient were considered comparable at baseline for both POSAS-PT (SUM *p* ≥ 0.266 for all, except FRF vs. control *p* = 0.031, OVERALL *p* ≥ 0.500) and POSAS-OBS (SUM *p* ≥ 0.281, OVERALL *p* ≥ 0.500).

#### Follow-up POSAS scores

At the 20-week FU, the patients perceived a general improvement (reduction of POSAS score) in their striae in all areas, being significant when compared to baseline on POSAS-PT with ΔSUM 5–10 points and ΔOVERALL 2–3 points. The point reduction in both SUM and OVERALL score was highest for the combination FRF + TT treatment. Only combination FRF + TT proved significantly better than the other areas of either monotherapy, TT or FRF, as well as untreated control (ΔSUM FRF + TT vs. TT: Δp = 0.033, FRF + TT vs. Control: Δp = 0.029, ΔOVERALL FRF + TT vs. Control, Δp = 0.041).

The blinded observer scores showed a tendency towards subtle improvement at 20-week follow-up versus baseline on POSAS-OBS with ΔSUM 0–2 points and ΔOVERALL 0–1 point. The combination treatment showed a tendency towards better improvement, but none of the interventions were significantly improved compared to each other or the untreated control (ΔSUM *p* ≥ 0.269, ΔOVERALL Δp ≥ 0.092 except for TT vs. Control Δp = 0.039 in favor of control).

**Fig. 2:**
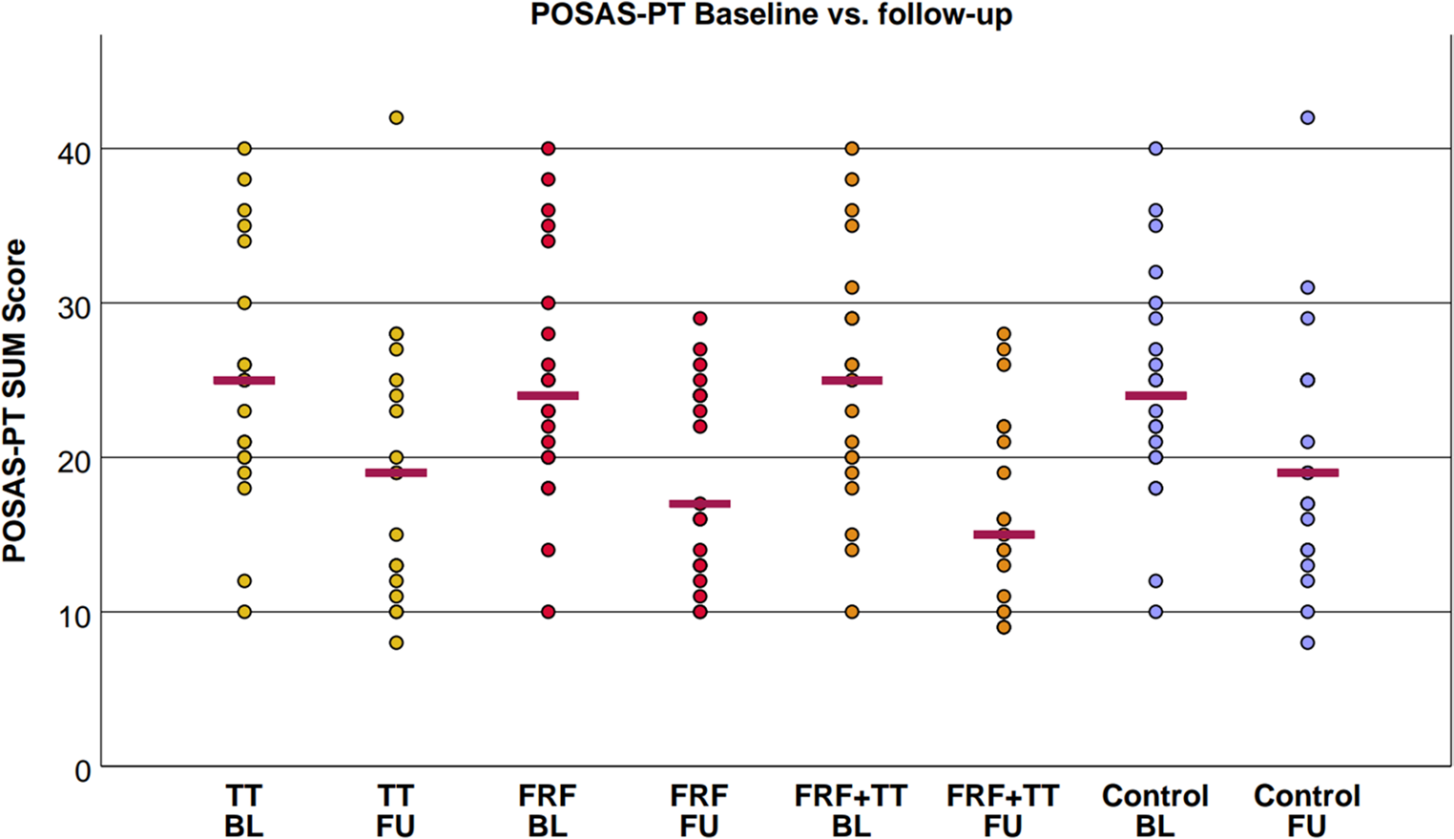
POSAS-PT SUM scores at baseline (BL) and at 20-week follow-up (FU) presented by group (TT, FRF, FRF + TT, Control) illustrated by medians and score distribution range, *n* = 19. Only FRF + TT BL vs. FU presented a significant difference compared to BL vs. FU in control group (Δp < 0.05)

See Fig. 2 for illustrated POSAS-PT and POSAS-OBS SUM data, respectively.

#### Correlations

The POSAS-PT and POSAS-OBS SUM improvement scores did not show a distinctive correlation with age or BMI (*Spearman’s correlation* *p* ≥ 0.061). The amount of used tretinoin did not show any correlation with improvement scores (*p* ≥ 0.151), for any of the relevant arms. A positive correlation between increasing treatment area size and observed improvement was found for the combination areas FRF + TT, specifically (POSAS-OBS SUM *p* = 0.03, OVERALL *p* = 0.002).

**Table 2:** POSAS-PT and POSAS-OBS SUM and OVERALL data from baseline to follow-up with corresponding p-values of paired test, *n* = 19

**TREATMENT**	**POSAS-PT**					
	**SUM Median (IQR)**			**OVERALL Median (IQR)**		
	**Baseline**	**Follow-up**	***P*-value** **BL vs. FU**	**Baseline**	**Follow-up**	***P*-value** **BL vs. FU**
TT	25 (20–33)	19 (12–25)	**0.008**	7 (5–8)	5 (3–7)	**< 0.001**
FRF	24 (20–33)	17 (13–24)	**0.002**	7 (4–8)	5 (3–6)	**0.001**
FRF + TT	25 (19–31)	15 (10–22)	0.003*	7 (4–9)	4 (3–6)	< 0.001*
Control (untreated)	24 (20–30)	19 (14–25)	**0.035**	7 (5–8)	5 (4–7)	**0.004**
	**POSAS-OBS**					
**TREATMENT**	**SUM Median (IQR)**			**OVERALL Median (IQR)**		
	**Baseline**	**Follow-up**	***P*-value** **BL vs. FU**	**Baseline**	**Follow-up**	***P*-value ** **BL vs. FU**
TT	12 (9–14)	11 (10–12)	0.058	3 (2–4)	3 (2–3)	0.148
FRF	12 (9–15)	12 (10–15)	0.288	3 (2–4)	2 (2–3)	**0.045**
FRF + TT	12 (8–15)	10 (9–14)	0.071	3 (2–4)	2 (2–3)	**0.005**
Control (untreated)	12 (8–14)	11 (10–12)	0.075	3 (2–4)	2 (2–3)	**0.003**

* Δp < 0.05 (significant difference compared to the BL vs. FU difference in control group)

#### POSAS SUM score items

The main improvements from baseline to follow-up upon which the patients reported in the SUM score were in *color* (*p* ≤ 0.006 for TT, FRF and FRF + TT, *p* = 0.071 for control), *thickness* (*p* ≤ 0.006 for TT and FRF + TT, *p* = 0.020 for FRF, *p* = 0.068 for control), *stiffness* (*p* = 0.043 for FRF + TT, *p* ≥ 0.339 for TT, FRF and control) and *irregularity* (*p* ≤ 0.004 for TT, FRF and FRF + TT, *p* = 0.011 for control). Comparing the different interventions, patients reported an improvement of *irregularity* (in favor of FRF + TT vs. TT: *p* = 0.032, in favor of FRF vs. Control: *p* = 0.055 (borderline)), *thickness (*in favor of FRF + TT vs. Control: *p* = 0.057 (borderline)), as well as *stiffness* (in favor of FRF + TT vs. TT: *p* = 0.041, FRF + TT vs. FRF: *p* = 0.007, FRF + TT vs. Control: *p* = 0.005, respectively). No other POSAS-PT SUM score items differed significantly between the interventions nor in comparison to the control (*p* ≥ 0.091).

The subtle improvements in the SUM score reported by the blinded observer were in *relief* (*p* = 0.047 for FRF + TT, *p* ≥ 0.063 for TT, FRF and control) and *pigmentation (p* = 0.035 for control, *p* ≥ 0.057 for TT, FRF and FRF + TT). No POSAS-OBS SUM score items differed significantly between each intervention nor in comparison to the control (*p* ≥ 0.378).

Considering the findings with appropriate statistical corrections (factor 6), differences within POSAS-PT SUM item improvements from baseline to follow-up were subtle in *color* and *irregularity* for TT, FRF, and FRF + TT and *thickness* for TT and FRF + TT. The *stiffness* improvement in favor of FRF + TT compared to FRF and control also remained significantly different. The remaining items were insignificant after correction and/or possibly random.

### Supporting measures

#### Exploratory visualization

**Fig. 3:**
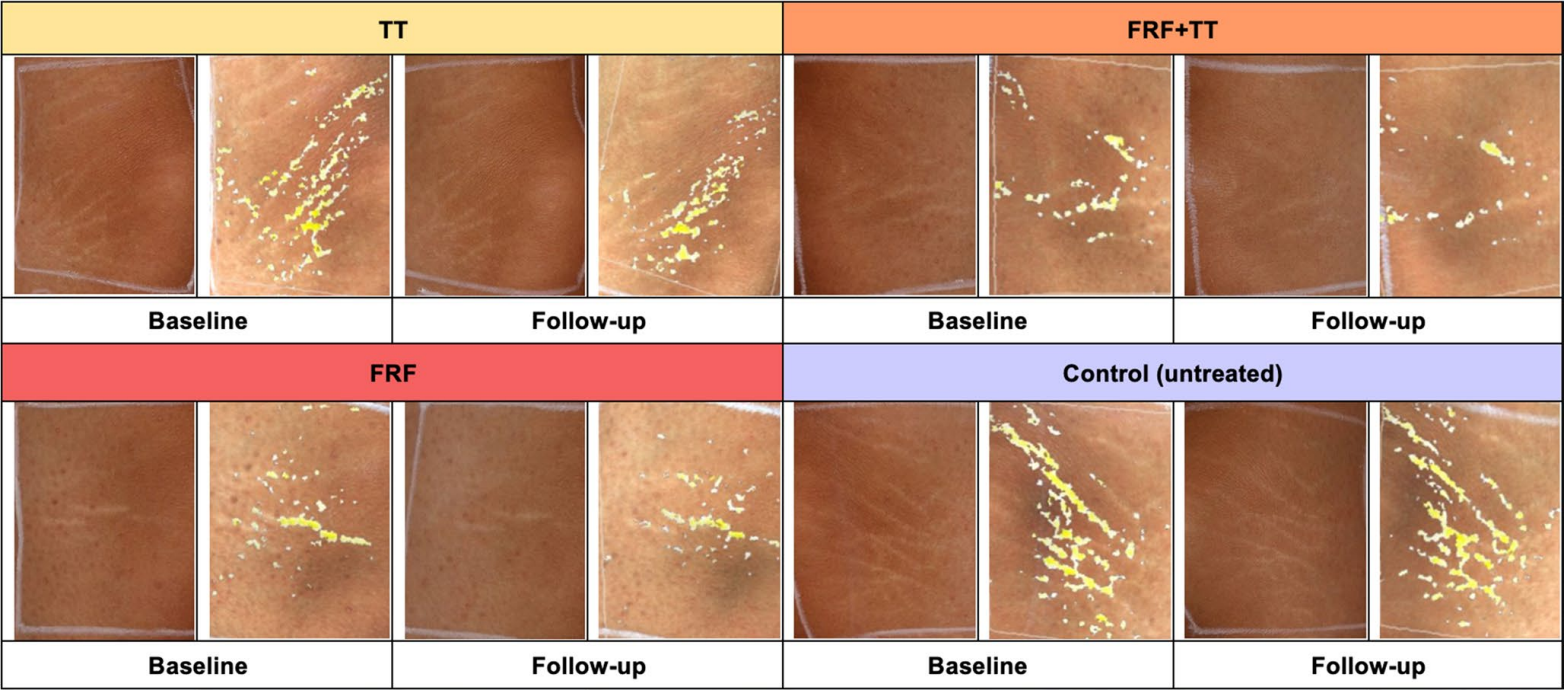
Example of visual improvement from combination FRF + TT, subtle improvement from TT and FRF, and no visual improvement in control; digital photos (left) and 3D images (right) with increasing depth of the lesions indicated with increasing yellow color intensity), *n* = 1

Visual changes were subtle and in general not easily captured with digital photography. Assessment of striae depth was attempted with the 3D camera but was inapplicable in the majority of patients due to the very superficial nature of some lesions. For illustrative purposes, Fig. 3 presents a patient with a visual response, particularly from the combination FRF + TT treatment, on POSAS-PT.

### Tolerability

Average procedural pain at baseline treatment with FRF was a median of 4 (IQR: 3–5) with a range between 2 and 8 and 80% being ≤ 5. While the experience of pain was rather individual, it was also associated with the anatomic site (*p* = 0.041), see Table 3. Three patients decided to proceed with local anesthesia for the following two treatments (2 inner thighs, 1 abdomen), but no patients withdrew due to procedural pain or any side effects.

**Table 3:** Procedural pain by anatomic site

Anatomic site, *n* (%)	Pain score, NRS median (range)
Abdomen, 9 (45%)	3 (2–7)
Buttocks, 4 (20%)	2.5 (2–3)
Inner thighs, 3 (15%)	4 (4–8)
Hips, 2 (10%)	4 (4–4)
Lower back, 1 (5%)	5 (.)
Knees, 1 (5%)	6 (.)

Common side effects were observed. Immediate skin reactions to FRF were redness and edema (100%, median 2 (IQR: 2–2)) that lasted up to one week. Light crusting after FRF was seen in some of the patients (32%, median 1 (IQR: 1–1)) and up to a few weeks, while some patients presented with light post-inflammatory hyperpigmentation (PIH) (21%, median 1 (IQR: 1–1)) that had decreased spontaneously by the end of study. In response to TT application, majority of patients experienced light intermittent rash or itch (80–89%, median 1 (IQR: 1–1.5), lasting between one week and one month, but none that limited the study procedures in following visits. See Table 4 for comparative data for TT and FRF + TT. None of the interventions gave rise to any significant downtime, and neither unexpected side effects nor serious adverse occurred during the study.

**Table 4:** Comparative tolerability data between TT and FRF + TT by number and percent, n (%)

Treatment	Erythema	Scaling/crusting	Dermatitis	PIH
TT	0 (0%)	0 (0%)	16 (80%)	0 (0%)
FRF + TT	19 (100%)	6 (32%)	17 (89%)	4 (21%)

#### Patient satisfaction

Patients were asked about satisfaction for each study areas at the 20-week follow-up visit. See Table 5 for overview of patient satisfaction rates. Satisfaction rates generally tended to be higher for the combination treatment.

**Table 5:** Patient satisfaction at end of study by intervention, *n* = 19

Satisfaction	TT	FRF	FRF + TT	Control
Very satisfied, *n* (%)	0 (0%)	0 (0%)	4 (21%)	0 (0%)
Satisfied, *n* (%)	2 (11%)	1 (5%)	5 (26%)	0 (0%)
Neutral, *n* (%)	16 (84%)	17 (90%)	8 (42%)	19 (100%)
Unsatisfied, *n* (%)	1 (5%)	1 (5%)	2 (11%)	0 (0%)
Very unsatisfied, *n* (%)	0 (0%)	0 (0%)	0 (0%)	0 (0%)

## Discussion

This randomized, controlled and GCP-monitored clinical trial examined the potential of combination treatment with FRF and TT for striae albae, which is a condition with unmet need for treatment options.

### Previous studies

Two studies have previously investigated the combination of FRF and TT [7, 18], but with significant differences to the present study.

In one study [18], FRF and TT (0.1%) combination was purely regarded as additive, as there was no actual FRF-assisted transdermal delivery of TT. Instead, two other formulations were applied immediately into the microthermal zones (MTZ) before subsequent TT application, limiting the assessment of a direct synergistic effect between FRF and TT. Additionally, both striae rubrae and albae were included, and only skin types III-IV, which was in direct contrast to the present study which included striae albae and skin types I-III only.

In another study [7], FRF and retinoic acid (0.05%) were combined in a small sample size of skin types III-IV, and delivery was actively enhanced with additional acoustic pressure ultrasound. Compared to the present study, both the skin types and the use of an additional enhancer differed between study designs. Furthermore, the prior study did not include randomization nor an untreated control group, which was in stark contrast to the present RCT design with intra-individual controls.

Both prior studies included histology. One study showed that combination of FRF and tretinoin increased the density of collagen and elastic fibers in the treated skin compared to untreated control [18]. The other study used ink formulation in a few histopathological samples and showed presences of ink in microchannels created with FRF [7]. While the present study did not include histology, it was based on a previously published pre-clinical paper showing that the specific utilized FRF-device had proven potential for topical uptake enhancement [17].

Altogether, the prior studies each supported the potential in combination treatment with FRF and TT, but each with their limitations. Both the background literature, and the related pre-clinical work, comprised the basis of the translational approach leading to the current RCT.

### Study main findings

In this side-by-side comparison with FRF monotherapy, TT monotherapy, and untreated control, the combination treatment performed better on patient-reported POSAS-scores. Although some improvements were also seen with TT and FRF monotherapy, overall, the study findings indicated a possible synergistic effect between FRF and subsequent TT. However, blinded observer POSAS-scores only showed a very subtle tendency in favor of the interventions, altogether, in comparison to the untreated control. In the POSAS-OBS data, there was a noticeable variance, and as such, a larger study population could have provided a clearer assessment pattern. Correlation of outcomes with treatment area size indicated that for larger areas such as abdomen and buttocks there was either a better treatment response or it was easier for the observer to clinically assess improvement.

The study was planned with a three-session treatment regime and with follow-up until 20 weeks from baseline to accommodate skin remodeling and neocollagenesis, which usually requires a matter of months to unfold and often multiple treatments before a significant clinical response. It is possible that additional treatments and/or a longer follow-up time could have unraveled more significant treatment responses, and hence improved the satisfaction rate.

### Strengths and limitations

The main strengths of this study were its stringent RCT and GCP-monitored clinical design as well as its translational approach, building on pre-clinical work that had verified the potential topical uptake enhancement of the used FRF-device.

However, there were limitations in the study. Firstly, the TT application, while individually monitored for total use of TT during the study, was not systematically monitored for exact compliance on day-to-day basis. While home-treatment remains a challenge to assess, this could have been facilitated further with additional user-friendly application schedules to be consecutively filled out by each participant. Secondly, the outcome measures in this study met several challenges in the assessment of striae. While POSAS is a well-established standardized scale for scars, it was not optimal for striae assessment. The single-blinded design increased the validity of the observer scores, but the baseline scores were particularly low even for substantially atrophic striae. This indicated that the objective scale items lacked affinity for particular features, such as the atrophy and fine, wrinkled, white appearance characterizing mature striae albae, compared to e.g., larger, hypertrophic and/or erythematous scars. The patient-reported scale evaluations contributed to the important assessment of patient perception of striae, but since blinding was not feasible, the risk of bias must be considered substantial. The discrepancy between patient and observer at both baseline and follow-up not only cements how striae substantially affect the individual but also emphasizes the importance of aligning treatment expectations based on the clinical reality.

While potential differences of treatment response relating to anatomical locations could have been interesting to identify and describe in detail, subgroup analysis was not feasible due to the heterogeneity in locations that did not provide comparable groups of acceptable sizes.

Finally, a main challenge in striae albae assessment included the clinical photographs, which could not visually capture the changes reported by patients on irregularity, thickness and stiffness. This was sought achieved with 3D images, however, even this technology struggled to capture the structural changes of striae albae, particularly of the more superficial lesions being clinically tangible only. Newer, updated device software could potentially improve the sensitivity to the superficial structural lesions.

With the recent, fast development of alternate imaging systems, the possibility to include better quantitative outcome measures, such as volumetric measures, for striae must be expected to improve.

## Future studies

In recent years, new assessment tools for striae have been under development [19–21], but objective assessment of striae albae remains to be definitively confirmed, which require extensive standardized validation studies, restricting a fast and easy implementation. In regard to general scar scales, POSAS has recently come out with a 3.0 version that allows for distinction of generic scars and linear, surgical scars. Whether this will improve the grading scale’s utility for striae albae remains to be investigated [22].

Objective measures that were not included in the given trial but could be considered in future studies for a continued translational approach could include assessment of dermal collagen, vascularity or pigmentation, skin texture, hydration and elasticity. On a cellular level, fibroblastic activity, cell proliferation, and inflammatory responses could be interesting to investigate.

Future treatment assessment may also consider a variety of factors and patient characteristics including skin type, age and BMI [23–26] Although this study did not find any, correlations could potentially be found in larger sample sizes with a wider representation.

### Perspectives

A continuous effort to establish new treatment approaches highlights the fact that striae lesions are some of the most undesirable, challenging lesions in skin. New and experimental treatments are on the rise, both including newer FRF devices, lasers, microneedling, ultrasound as well as new formulations such as platelet-rich plasma and fillers [16–33]. The potential of combination treatments is vast and may further expand the field of energy-based device-assisted topical delivery.

Despite studies reporting promising results, the generalizability suffers greatly due to an extensive heterogeneity in striae assessment; different objective assessment tools, patient-reported outcome measures, and even visualization of striae. There is a lack of high-quality clinical trials that allow direct comparison of different treatments, however, the body of evidence is persistently growing with new pilot studies with EBDs and newer evaluation tools, showing promising results [34]. Additionally, increasing numbers of high-quality clinical trials will enable the possibility for systematic review providing an overview and comparison of different treatment approaches and potential future directions. With further development in the field, the possibility of increasingly individualized approaches based on skin quality, body type, skin color, and even patient preference will be made possible.

## Conclusion

Based on the patient-reported responses, treatment with a combination of FRF and TT improved striae albae compared to the untreated control. The features that patients reported noticeable improved following combination treatment, in particular, were irregularity, thickness, and stiffness of their striae.

Therapy for striae albae remains immensely challenging, but the findings suggest a potential for future combination treatment approaches that may enhance the patients’ perception and overall opinion of their striae.

## Data Availability

The data presented in this study is available upon reasonable request.

## Abbreviations

BL: Baseline
BMI: Body Mass Index
FU: Follow-up
GCP: Good Clinical Practice
IQR: Interquartile Range
NRS: Numerical Rating Scale
SA: striae albae
SR: striae rubrae
OBS: observer
PIH: post-inflammatory hyperpigmentation
POSAS: Patient Observer Scar Assessment Scale
Pt: patient
u-hCG: Urine human chorionic gonadotropin
W: week
